# First-Principles Study on the Adsorption Behavior of O_2_ on the Surface of Plutonium Gallium System

**DOI:** 10.3390/ma15145035

**Published:** 2022-07-20

**Authors:** Longxian Li, Min Zhu, Guikai Zheng, Yan Li, Yang Yang, Yilong Liu, Huan Su

**Affiliations:** 1 College of Nuclear Science and Technology, Naval University of Engineering, Wuhan 430000, China; lilongxian0623@126.com (L.L.); yacareft@163.com (Y.L.); 13547912889@163.com (Y.Y.); wadeview@163.com (Y.L.); sususuhuan@163.com (H.S.); 2 Unit 93671 of the Chinese People’s Liberation Army, Nanyang 473000, China

**Keywords:** surface adsorption, first principles, plutonium gallium alloy, the electronic structure

## Abstract

To deeply understand the adsorption process of oxygen on the surface of a plutonium gallium system and to reveal the chemical reaction mechanism at the initial stage of oxidative corrosion on the surface of plutonium gallium alloy at a theoretical level, the adsorption behavior of oxygen molecules on the surface of a plutonium gallium system was investigated by a first-principles approach based on density flooding theory. The results show that the molecular bond length increases and finally breaks when the surface oxygen molecule is adsorbed on the surface of plutonium gallium system and dissociates into two atomic states. The most likely adsorption position of oxygen molecules on the surface of plutonium gallium system is hole-site vertical adsorption with the adsorption energy size of 10.7 eV. The bonding between oxygen atom and surface is mainly due to the overlapping hybridization of Pu-6s, Pu-7s, Pu-6d, Ga-3d and O-2p orbitals. Oxygen molecules mainly interact with the atoms of the first layer on the surface of the plutonium gallium system. The oxygen atoms after stable adsorption are able to diffuse to the subsurface of the plutonium gallium system after overcoming the energy barrier of 16.7 eV and form a stable structure. The research results reveal the initial reaction process and adsorption law of oxygen on the surface of plutonium gallium system from microscopic level, which is helpful to further explore the surface corrosion prevention technology of plutonium gallium system and improve the reliability and safety of plutonium gallium alloy components.

## 1. Introduction

Plutonium is one of the most complex elements in the periodic table and has many unique properties [1,2]. Its electronic properties are extremely complex, with exotic f-f electron interactions and apparent relativistic interactions [3,4]. The doping of gallium element can increase the temperature stability of δ-plutonium, while significantly improving the flexibility and ductility of the metal, so most of the practical applications in the nuclear industry are plutonium gallium alloys. Plutonium-based materials are very chemically active and can easily react with hydrogen, oxygen and water vapor in the environment during storage and placement, causing surface corrosion [5,6]. This poses a significant challenge for the long-term storage and protection of plutonium-based materials. The adsorption behavior of oxygen molecules on plutonium-based materials is the primary process by which oxidative corrosion occurs on the surface. Therefore, studying the adsorption process of oxygen molecules on plutonium-based surfaces is important for gaining insight into the corrosion and corrosion prevention mechanisms of plutonium-based materials. In fact, plutonium-based materials are highly radioactive and toxic, and direct experimental studies are rarely performed. The density flooding theory (DFT) method is currently the dominant computational method in condensed matter physics and a reliable method for studying the properties of plutonium alloys and their compounds.

In terms of theoretical calculations of Pu-Ga alloys, Sadigh et al. [7] studied the structural and thermodynamic properties of δ-phase Pu-Ga alloys using a plane-wave pseudopotential method based on Spin Polarized (SP) density generalized theory (DFT), and the results showed that the full structural properties and most thermodynamic properties of low Ga content δ-phase Pu-Ga alloys can be successfully calculated by considering the spin polarization effect. Luo Wenhua [8] et al. used the full potential line affixation plus plane wave (FPLAW) method to calculate the equilibrium structure, electronic structure and heat of formation of alloying elements such as Ga and Pu compounds under the generalized gradient approximation (GGA) + spin-orbit coupling (SOC) + SP conditions, and the results showed that the Pu and alloying elements The hybridization between atomic orbitals depends on the competition between 6d and 5f of Pu atoms, *p* of alloyed atoms and 6d of Pu atoms, and s and *p* orbital hybridization of alloyed atoms. Regarding the adsorption process of oxygen molecules on the plutonium surface, Huda et al. [9] studied the adsorption of individual oxygen atoms on the δ-Pu (100) and (111) surfaces using the generalized gradient approximation of DFT using DMol3 software. The central positions of the δ-Pu (100) and δ-Pu (111) surfaces are the most favorable locations for adsorption under spin-polarized conditions with chemisorption energies of 7.386 eV and 7.070 eV, respectively. This author [10] also investigated in detail the adsorption of oxygen molecules on the δ-Pu (100) surface using the same approach. It was found that oxygen molecules tend to dissociate for adsorption compared to single-atom adsorption. Among all the adsorption configurations studied, the parallel direction on the bridge position was the best adsorption site with the highest energy, with an adsorption energy of 8.787 eV. Wei Hongyuan et al. [11] The adsorption behavior of oxygen atoms on the δ-Pu (111) surface was investigated using the DMol3 program using GGA-DET combined with the rPBE function. The results showed that the type of adsorption was a strong chemisorption, and the main factor determining the chemisorption was the coordination number of Pu and O. The chemical bonding became more and more stable with the increase in coordination number, and the interaction between the plutonium surface and oxygen atoms occurred mainly in the first layer The interaction between the plutonium surface and oxygen atoms mainly occurs in the first layer, and the influence of the remaining two layers is very small [12]. The adsorption behavior of oxygen molecules on the surface of δ-Pu (100) was studied by GGA-DET method. The results showed that after the adsorption of O2 on the surface of δ-Pu (100), the O-O bond length increased until it broke, and dissociated into two atoms adsorbed on the surface of δ-Pu (100), and the dissociated O atoms preferentially occupied the cavity and bridge sites, and the most stable adsorption conformation adsorption energy was 8.14 eV. Currently, regarding the adsorption of oxygen on the surface of plutonium gallium alloys Hernandez et al. [13] used the WIEN2k program to calculate the adsorption of oxygen atoms on the stable δ-Pu (111) surface of gallium using the (FP-L/APW + lo) method. The results show that the hollow fcc position is the most prone to adsorption with an adsorption energy of 4.98 eV. The oxygen atoms tend to adsorb on the surface and do not easily diffuse to the interior. The adsorption of oxygen atoms can cause a small change in the work function.

In view of the above, a number of theoretical calculations on the interaction between oxygen and plutonium gallium alloy have been published in recent years, mostly before 2015. It can be seen that most theoretical calculations have focused on the plutonium gallium alloy properties and the adsorption of oxygen atoms or oxygen molecules on the pure plutonium surface, and the adsorption on the plutonium gallium alloy surface is less studied. At present, the adsorption of oxygen molecules on the surface of plutonium gallium system using VASP procedure has not been reported. Therefore, in this paper, we establish a doping model of plutonium gallium system for δ-Pu (100) surface doped gallium atoms and perform first-principles calculations on the adsorption of oxygen molecules on the surface of plutonium gallium system. We focus on the analysis of the interaction of oxygen with the surface of the plutonium gallium system in terms of the adsorption configuration, adsorption energy, electronic structure change and charge transfer for stable adsorption, and we apply the transition state search algorithm to calculate the potential barrier for the diffusion of oxygen atoms to the interior of the surface. Our study provides a theoretical basis for the corrosion and anti-corrosion mechanism of oxygen on the surface of plutonium gallium alloy.

## 2. Calculation Methods and Models

### 2.1. Calculation Method

All calculations were performed using the first-principles-based VASP [14,15] (Vienna ab initio simulation package) software. The valence electron wave function is expanded with a projector augmented wave (PAW) [16,17], projector augmented wave (PAW) expansion, and the exchange-correlation generalized gradient approximation (GGA) [18]. The exchange-correlation generalization is handled by the Perdew-Burke-Ernzerhof (PBE) approximation in the framework of generalized gradient approximation (GGA). The inner electrons and nuclei are replaced by first-principles pseudopotentials, where the valence electrons of Pu are described by 6s^2^6p^6^6d^2^5f^4^7s^2^, Ga by 4s^2^4p^1^, and O by 2s^2^2p^4^. The Monkhorst-Pack [19] (MP) method is used to generate the k-point grid of Brillouin zone integrals. For the optimization calculations of the adsorption configuration in this paper, 5 × 5 × 1 is used. During the optimization process, the plane wave truncation energy is set to 520 eV and the electron self-consistency calculation convergence accuracy is set to 1 × 10^−5^ eV. The optimization is considered to be completed when the residual stress of the adsorption configuration is less than 1 × 10^−2^ eV/nm. The spin polarization effect was considered in all calculations.

The adsorption energy *E*_ads_ of oxygen molecules on the surface of the plutonium gallium system is defined by the following equation

(1) $$E_{\mathrm{ads}}=E_{\left(surface+O_{2}\right)}-E_{\left(O_{2}\right)}-E_{\left(surface\right)}$$

where $E_{\left(surface+O_{2}\right)}$ is the energy of the optimized adsorption configuration, $E_{\left(O_{2}\right)}$ is the energy of the adsorbed molecule optimized separately before adsorption, $E_{\left(surface\right)}$ is the energy of the surface of the plutonium gallium system optimized separately before adsorption, and each energy on the right side of the equation is the energy optimized in the same way and with the same precision. According to the definition of Equation (1), the negative value of the adsorption energy means that the adsorption is an exothermic reaction, and the larger value indicates stronger adsorption and higher adsorption stability.

### 2.2. Computational Models

**Figure 1:**
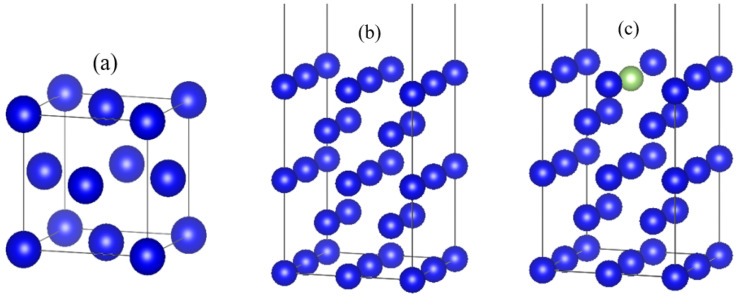
(**a**) Single cell structure. (**b**) Surface model. (**c**) Plutonium gallium system model (The blue spheres represent plutonium atoms, and the green spheres represent gallium atoms).

The crystal structure of δ-Pu metal is face-centered cubic (fcc) with space group code Fm-3m, the experimental values of lattice constants are a = b = c = 4.637 Å [20] and α = β = γ = 90°, the optimized lattice constant is calculated as a = b = c = 4.776 Å, α = β = γ = 90°, volume V = 108.9489 Å^3^ and bulk phase energy E = −54.731 eV. The error between the lattice constant and the experimental value is 2.9%, which indicates good agreement, and the results of single cell optimization are shown in Figure 1a. The optimized single cell is faceted, and the surface model is used to establish the crystallographic surface model of δ-Pu using the (100) surface. The atomic structure of 5 layers is selected, and the thickness of the vacuum layer is set to 15 Å. The structure of the crystal surface is shown in Figure 1b. The surface-most atoms have a large influence on the surface properties, and four different doping models with different gallium atomic contents are established by substituting different numbers of gallium atoms on the surface-most face of the doped δ-Pu (100). The doping form with the smallest and most stable surface energy, i.e., gallium atom doping, replaces the most central plutonium atom on the surface, is obtained by calculation, as shown in Figure 1c. This surface model contains 20 atoms, of which 19 are plutonium atoms and 1 is a gallium atom.

**Figure 2:**
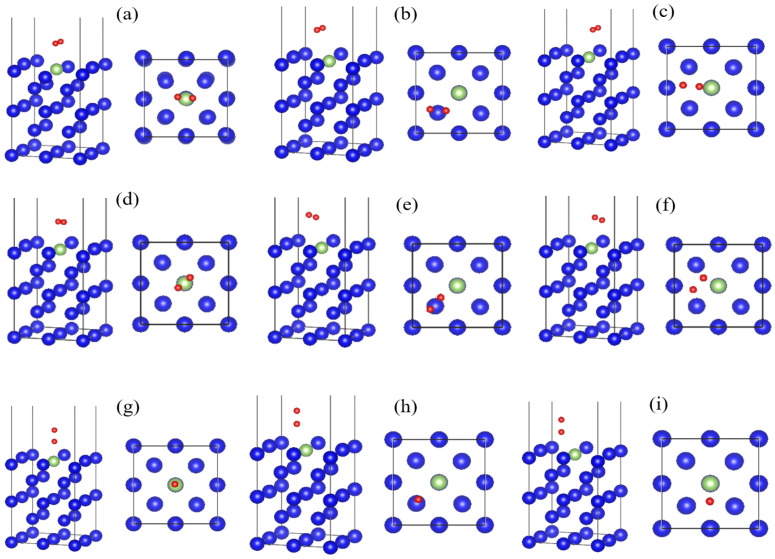
Different initial adsorption configurations of O_2_ on the surface of plutonium gallium system: (**a**) O-t-hor1, (**b**) O-h-hor1, (**c**) O-b-hor1, (**d**) O-t-hor2, (**e**) O-h-hor2, (**f**) O-b-hor2, (**g**) O-t-ver, (**h**) O-h-ver, (**i**) O-b-ver. (O: oxygen molecule; t: top position; h: heart position; b: bridge position. The red sphere is oxygen).

In the construction of the adsorption model, a hydrogen molecule is placed on the surface of an optimized plutonium gallium system containing 19 plutonium atoms and 1 gallium atom with a coverage of 0.25. The bond length of H-H in H_2_ is 0.7372 Å. The adsorption molecules are placed on the bridge, hollow and top positions of the plutonium gallium system. molecules in each adsorption position are considered three adsorption directions: adsorption molecules are perpendicular to the surface (ver); adsorption molecules are parallel to the surface and parallel to the crystal axis (hor1); adsorption molecules are parallel to the surface and parallel to the diagonal of the crystal axis (hor2). All nine initial adsorption configurations of O_2_ are on the surface of the plutonium gallium system, as shown in Figure 2. For example, O in O-h-ver represents the adsorption of hydrogen molecules, h indicates that the initial adsorption position is a hole site (hollow), and ver indicates that the gas molecules are placed perpendicular to the surface.

## 3. Results and Discussion

### 3.1. Adsorption Conformation and Adsorption Energy

**Figure 3:**
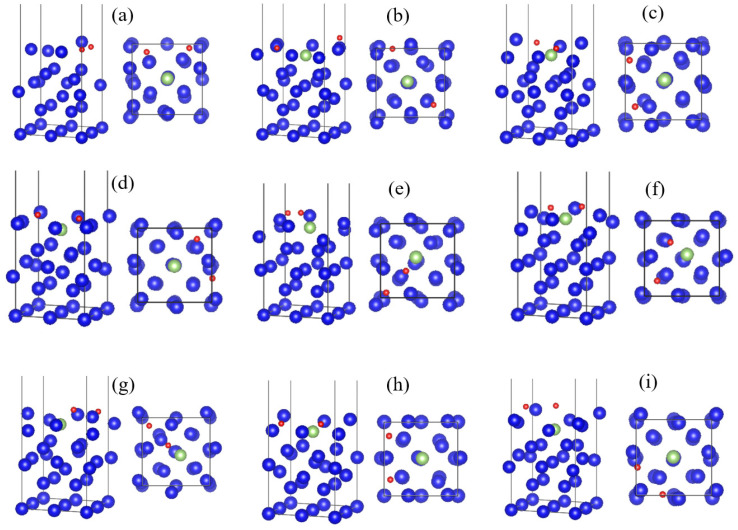
Stable adsorption configuration of O_2_ on the surface of plutonium gallium system after optimization: (**a**) O-t-hor1, (**b**) O-h-hor1, (**c**) O-b-hor1, (**d**) O-t-hor2, (**e**) O-h-hor2, (**f**) O-b-hor2, (**g**) O-t-ver, (**h**) O-h-ver, (**i**) O-b-ver.

The nine adsorption configurations of O_2_ on the surface of plutonium gallium system are optimized, and the structural parameters and energy data of oxygen adsorption on the surface of plutonium gallium system are given in Table 1, and Figure 3 shows the stable adsorption configurations of O_2_ on the surface of plutonium gallium system after structural optimization. From the data in Table 1, it can be analyzed that the adsorption energies of the nine initial adsorption configurations of oxygen at the core, bridge and top positions are all negative, and all of them can undergo stable adsorption, and the adsorption energies of their various adsorption configurations are between −5 and −10 eV, which give off a large amount of heat. From the spacing between oxygen atoms after adsorption, the bond length of oxygen molecules before adsorption is 1.23 Å, and the spacing between two oxygen atoms after adsorption is much larger than the initial bond length, which indicates that oxygen molecules are dissociated during the process of adsorption, and the distance between oxygen atoms and the surface after adsorption is within 1 Å. The O-O bond is broken to form two oxygen atoms adsorbed on the surface of plutonium gallium system, and new chemical bonds are formed with the surface plutonium and gallium atoms. From the adsorption position, the oxygen molecule corresponds to a larger adsorption energy at the hole position, followed by the bridge position, and at the top position is the smallest adsorption energy, and the three forms of oxygen itself have less influence on the adsorption energy. The adsorption stability of oxygen on the surface of plutonium gallium system is in the order of hollow position > bridge position > top position. By observing the optimized stable adsorption configuration, it can be seen that the process of oxygen adsorption, the surface of plutonium gallium system undergoes a greater degree of structural changes, indicating that there is a greater degree of interaction between oxygen and plutonium gallium surface.

These phenomena show that oxygen is easily adsorbed onto the surface of the plutonium gallium system, and all oxygen molecules are able to dissociate into two oxygen atoms adsorbed on the surface and form new chemical bonds with the surface, which is chemisorption.

### 3.2. Bader Charge Analysis

The adsorption reaction of oxygen on the surface of the plutonium gallium system is necessarily accompanied by the transfer of charge between the active gas molecules and the surface atoms of the plutonium gallium system. The Bader charge analysis can be used to quantitatively represent the amount of charge transfer and analyze the surface charge transfer. Table 2 shows the amount of transferred charges for each layer of atoms and the adsorbed gas atoms for each stable configuration.

Analysis of the data in the table shows that the oxygen molecules all get charge during the adsorption process, and the amount of charge transferred is above 2e, which indicates that the oxygen has a large charge transfer with the surface atoms and a large interaction, which also indicates that the oxygen is chemisorbed on the surface. Among the various stable configurations of oxygen, the charge transfer amounts do not differ much, indicating that the adsorption position has a small effect on the charge transfer. Among all the stable adsorption configurations, the atoms of each layer of the plutonium gallium system surface generally show that the atoms of the first, second and fifth layers lose charge and the atoms of the third layer gain charge. In terms of numerical magnitude, the charge transfer of the first layer is the largest, all above 2e, and the charge transfer of the atoms of the remaining layers is below 1e, indicating that the gas molecules mainly interact with the atoms of the first layer of the surface.

In general, the adsorption of oxygen on the surface of the plutonium gallium system is accompanied by a large degree of charge transfer in the direction of charge transfer from the surface of the plutonium gallium system to the oxygen molecules, and the charge transfer occurs mainly in the first layer of atoms on the surface.

### 3.3. Electronic Structure Analysis

#### 3.3.1. Differential Charge Density Analysis

The differential charge density is the difference between the charge density after bonding and the atomic charge density at the corresponding point. By calculating and analyzing the differential charge density, the properties of charge movement during bonding and bonding electron coupling as well as the direction of bonding polarization can be clearly obtained, which helps to understand the process of adsorption.

Differential charge density.

(2) $$\Delta \rho =\rho_{A+B}-\rho_{A}-\rho_{B}$$

where Δρ is the differential charge density value, $\rho_{A+B}$ is the charge density value of the adsorbed configuration, $\rho_{A}$ is the charge density value of the adsorbed molecules after adsorption, and $\rho_{B}$ is the charge density on the surface of the plutonium gallium system after adsorption.

**Figure 4:**
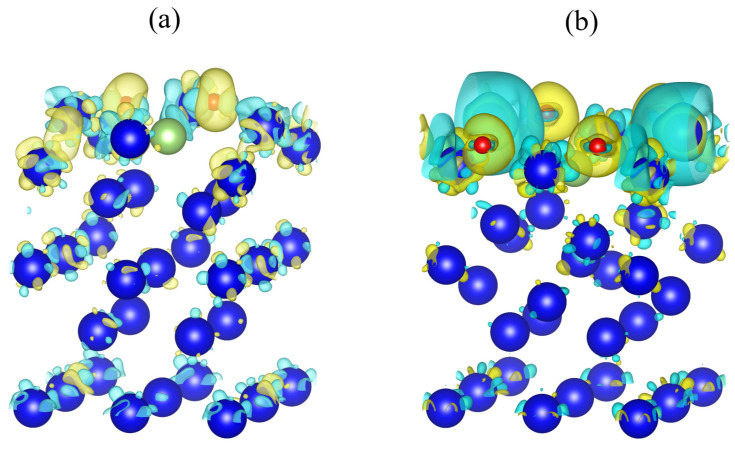
Differential charge density: (**a**) O-b-hor2 (**b**) O-h-ver. (Note: the electron density equivalent surface is 7.0 e/nm^3^, yellow indicates an increase in charge density, blue indicates a decrease in charge density).

The hole-site vertical adsorption with higher adsorption energy and the bridge-site parallel adsorption with lower adsorption energy were selected to demonstrate the differential charge density, using the same electron density equivalent surface. The differential charge density diagram of O-b-hor2 and O-h-ver configurations is shown in Figure 4. In both adsorption configurations, the oxygen atom is shown in yellow around the oxygen atom, indicating an increase in charge density near the oxygen atom and a decrease in charge density around the neighboring plutonium atom near the oxygen atom into blue. The charge transfer is the greatest in the first layer among the atoms on the surface of the plutonium gallium system, and it can be observed from the differential charge density diagram that the oxygen atom mainly interacts with the atoms in the first layer on the surface, which is consistent with the Bader charge calculation. Comparing the differences between the two configurations, it can be seen that the O-h-ver configuration with a larger adsorption energy has a larger degree of charge transfer, while the O-b-hor2 configuration with a smaller adsorption energy has a smaller degree of charge transfer, which indicates that a larger degree of charge transfer and a larger degree of interatomic interaction tend to give off more heat and form a more stable adsorption configuration.

#### 3.3.2. Electron Density of States Analysis

**Figure 5:**
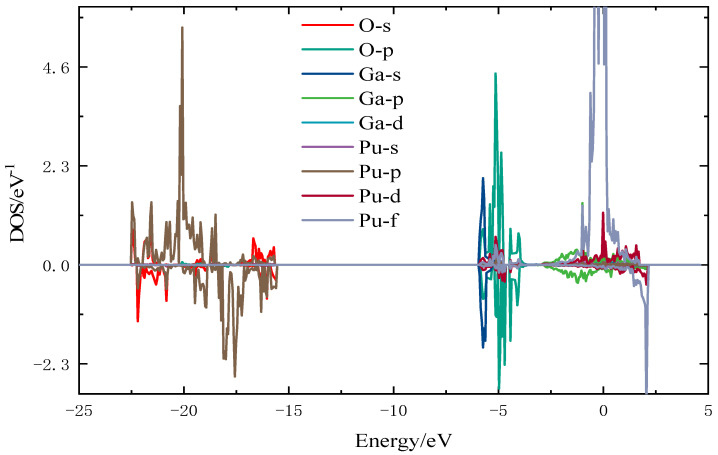
Fractional wave density of states for O-h-ver configuration.

**Figure 6:**
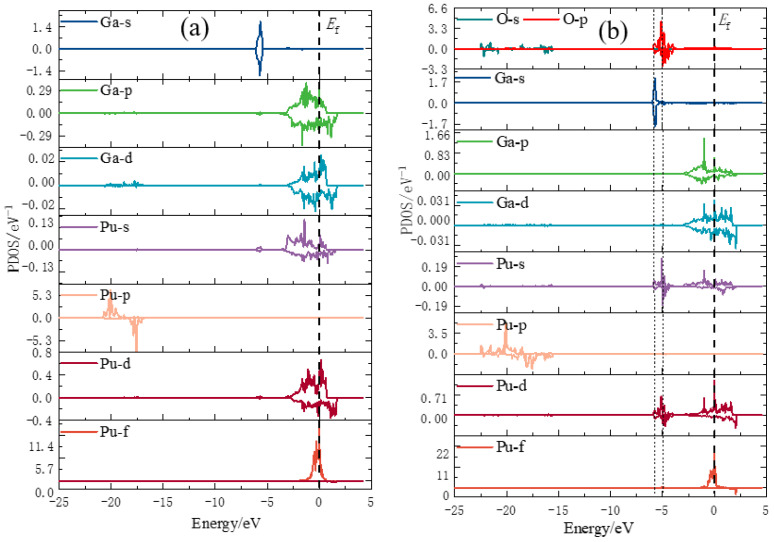
Fractional density of oxygen molecules after adsorption on the surface of plutonium gallium system: (**a**) fractional density of pure surfaces; (**b**) fractional density of oxygen after adsorption.

To further investigate the microscopic interactions between oxygen atoms and surface atoms of the plutonium gallium system, the most stable configuration for oxygen adsorption (O-h-ver) was selected, and the density of states was calculated and analyzed. The density of states is shown in Figure 5 and Figure 6, where an oxygen atom and the nearest plutonium and gallium atoms are selected.

As can be seen in Figure 5, the density of states of the stable adsorption configuration is mainly distributed in three regions, with Pu-5f, Pu-6d and Ga-4p orbitals near the Fermi energy level, O-2p and Ga-4s orbitals near −5 eV, and O-2s and Pu-6p orbitals occupying mainly near −23 eV–15 eV.

The microscopic mechanism of chemisorption between adsorbed atoms and the surface can be analyzed by comparing and analyzing the changes of surface state density before and after adsorption in Figure 6. Firstly, considering the interaction between oxygen and plutonium atoms, near −5 eV, the electronic states of the 6s, 7s and 6d orbitals of the plutonium atoms are cleaved, and new obvious spikes with larger peaks appear, indicating that hybridization between O-2p and Pu-6s, Pu-7s and Pu-6d orbitals occurs, forming a stable bonding interaction. Near −24 eV to −16 eV, the peak of Pu-6p orbital decreases, and the spreading increases, and is basically the same as the spreading of O 2s orbital, and the other orbital electronic states of Pu atoms do not change to a larger extent near this energy. Secondly, considering the interaction between oxygen atom and gallium atom, a spike of smaller intensity appears in the 4s orbital of gallium atom near −5 eV, and the spreading of 3d orbital of gallium atom also increases to some extent near −24 eV to −16 eV, but it is not significant. During the adsorption of oxygen molecules on the surface of plutonium gallium system, electron transfer occurs in the atomic orbitals of both oxygen molecules and plutonium gallium system. The interaction between plutonium atoms and oxygens atom is stronger, and the interaction between plutonium atoms and oxygen atoms is mainly due to the overlapping hybridization of Pu-6s, Pu-7S, Pu-6d, Ga-3d and O-2p orbitals.

### 3.4. Surface Power Function Analysis

The work function is the minimum energy required to move an electron from the interior of a solid to the surface. It is the energy difference between the vacuum electrostatic potential and the Fermi energy level at an infinite distance from the outside of the metal, and represents the ability of electrons to escape to the surface of the metal, calculated as follows.

(3) $$\Phi =E_{\mathrm{vacuum}}-E_{\mathrm{fermi}}$$

where Φ, $E_{\mathrm{vacuum}}$ and $E_{\mathrm{fermi}}$ denote the work function, vacuum level and Fermi level, respectively, in eV.

We analyzed the changes of the surface work function of the pure Pu-Ga system and the surface work function before and after the adsorption of oxygen molecules, and the calculated results are shown in Table 3. There are no experimental data available on the surface function of Pu, and the work function of its neighboring element uranium is in the range of 3.63 eV–3.90 eV [21]. Wei Hongyuan et al. [22] calculated the work function for the bare surface of δ-Pu (100) as 4.365 eV, and Atta-Fynn et al. [23] calculated the work function of δ-Pu (111) slab as 3.39 eV, and the surface work function of the plutonium gallium system calculated in this paper is 2.9895 eV based on the substitution of δ-Pu surface doped with certain Ga atoms, which is reasonable considering the difference of calculation methods and calculation models.

**Figure 7:**
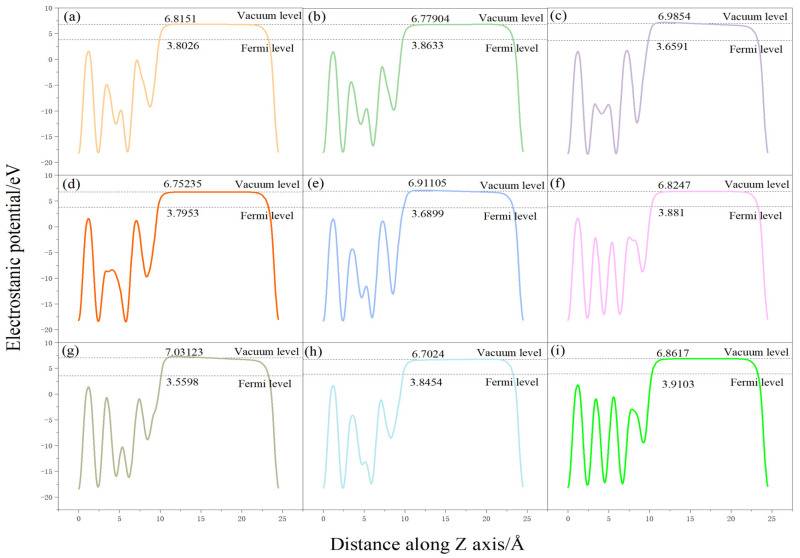
Variation of electrostatic potential along *Z*-axis for various configurations: (**a**) O-t-ver, (**b**) O-t-hor1, (**c**) O-t-hor2, (**d**) O-h-ver, (**e**) O-h-hor1, (**f**) O-h-hor2, (**g**) O-b-ver, (**h**) O-b-hor1, (**i**) O-b-hor2.

After oxygen adsorption, the surface work function appears to change to some extent; most of the stable conformation work function changes are small. The work function of each configuration after adsorption is shown in Figure 7. There are two conformations O-b-ver and O-t-hor2, and the conformation work function increased by 0.4819 eV and 0.3368 eV, respectively. A stable adsorption conformation can be observed, and it was found that most of the surface atoms had a certain degree of displacement, causing surface undulation. When analyzing the reason for the change of surface work function, due to the adsorption of oxygen on the surface of plutonium gallium system, a surface dipole moment from the surface to the oxygen molecule is formed on the surface, and the work function appears to vary to different degrees due to the different factors such as adsorption distance and transferred charge. The corresponding work function changes are also larger for large changes in surface atomic configuration.

### 3.5. Diffusion of Oxygen into the Interior of the Plutonium Gallium System

The analysis above shows that oxygen spontaneously dissociates during surface adsorption, and the O-O bond breaks to form two oxygen atoms chemically adsorbed on the surface. Oxygen is capable of oxygen etching on the surface of the plutonium gallium system, which is inevitably accompanied by the diffusion of oxygen atoms to the interior of the surface, and we next studied the diffusion of oxygen atoms to the interior of the surface after adsorption on the surface.

**Figure 8:**
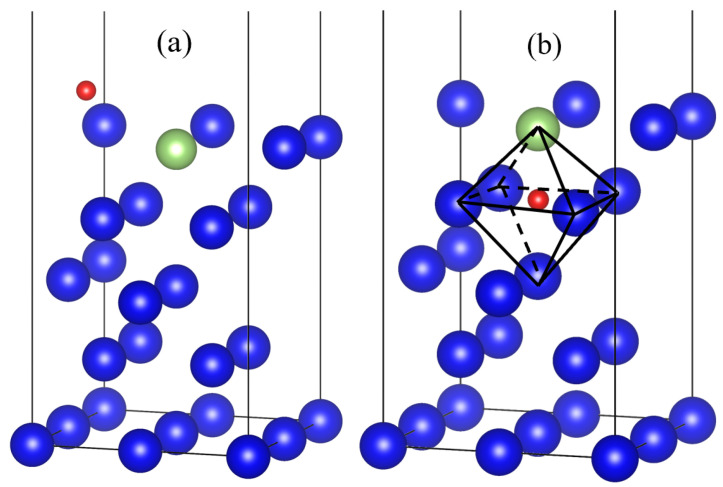
Transition state calculation of initial and final states: (**a**) initial state; (**b**) final state.

To simplify the problem, an oxygen atom and plutonium gallium system surface is selected as the study object, the structure optimization of the initial and final states is carried out, the intermediate state model is constructed, and the transition state search is based on [24,25]. The calculation is based on the transition state search algorithm. The stable configuration of the oxygen atom after adsorption on the surface is chosen for the initial state, as shown in Figure 8a. For the end-state structure, the oxygen atom is chosen to be placed in the second layer of the surface, and the initial state is placed in the middle position of the two plutonium atoms in the second layer. During the structure optimization, the oxygen atom is spontaneously displaced to the four atomic centers, i.e., the octahedral center, which represents that the oxygen atom has a more stable structure in the octahedral center.

**Figure 9:**
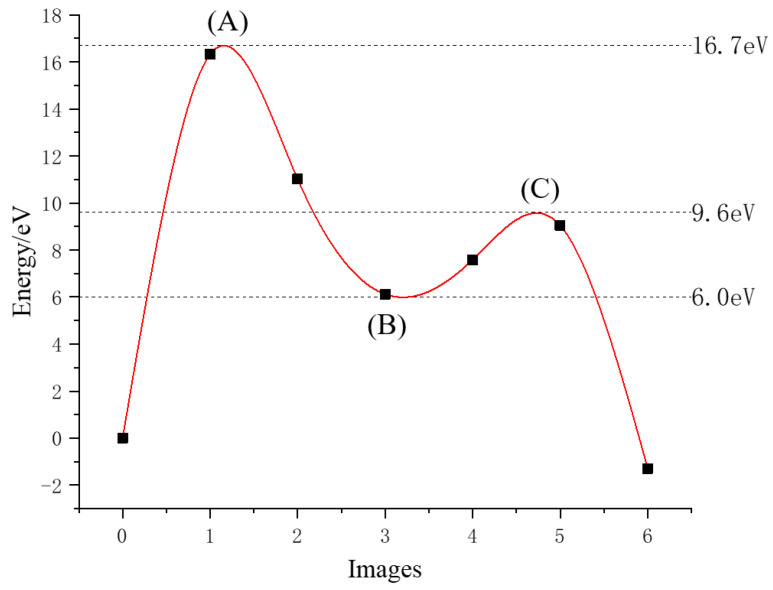
Minimum energy path for oxygen atom diffusion.

**Figure 10:**
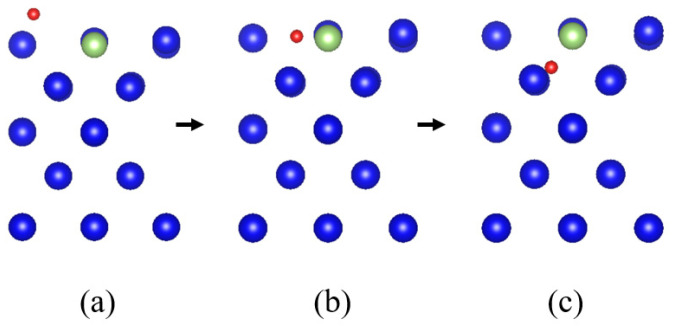
Intermediate state of diffusion process: (**a**) image1, (**b**) image3, (**c**) image5.

The minimum energy path for the diffusion of hydrogen atoms to the interior of the surface of the plutonium gallium system is calculated as shown in Figure 9. As the oxygen atoms spread inward, they continue to move closer to the surface, overcoming an energy barrier of 16.7 eV, as shown at point (A) in Figure 9. After crossing the energy potential barrier, the oxygen atoms continue to approach the surface until the oxygen atom is embedded in the first layer of atoms on the surface, located between four adjacent atoms, at which point the point of minimal energy value is reached, indicating that the oxygen atom can be stably embedded in the hole position of the first layer of atoms. If the oxygen atom continues to diffuse to the second layer of atoms, it needs to overcome the energy potential barrier of 3.6 eV at this point, cross the point (C), and finally enter the center of the four atoms in the second layer to complete the diffusion process. The intermediate states of the diffusion process are shown in Figure 10. Figure 10a represents the state of the oxygen atom when it is close to the first layer of the surface, Figure 10b represents the very small value of energy in the diffusion path where the oxygen atom is exactly embedded in the first layer of atoms, and Figure 10c represents the state of the oxygen atom when it is close to the second layer of atoms on the surface.

## 4. Conclusions

At present, most theoretical calculations of plutonium surface adsorption do not consider the doping of gallium atoms, which is different from the actual situation. The adsorption behavior of oxygen molecules on the surface of plutonium gallium system was calculated and analyzed using a first-principles method. The main findings of the paper are as follows:

(1) Oxygen molecules dissociate and adsorb on the surface of the plutonium gallium system without overcoming the energy barrier. The O-O bond in the oxygen molecule breaks and dissociates into two oxygen atoms attached to the surface. The best adsorption position was vertical adsorption in the hole, and the adsorption energy was 10.7 eV. The order of oxygen adsorption stability on the surface of plutonium gallium system is hole position > bridge position > top position.
(2) Bader charge calculation results show that oxygen molecules gain charge (2.2618 e–2.4617 e) during the adsorption process, mainly by interacting with the first layer of atoms on the surface. Electron structure analysis shows that the reaction mechanism between oxygen molecules and surface atoms of plutonium gallium system is that new chemical bonds are generated by hybridization of 2p orbital of oxygen atom with 6s, 7s, 6d orbital of plutonium atom and 3d orbital of gallium atom.
(3) After oxygen molecules are dissociated and chemically adsorbed on the surface, they can diffuse to the sub-surface atomic layer on the surface of the plutonium gallium system after surmounting the energy barrier of 16.7 eV, and form a stable structure. This indicates that after oxygen molecules are dissociated and adsorbed, they can continue to diffuse inward under certain energy, which changes the surface structure.

The adsorption behavior of oxygen molecules on the surface of plutonium gallium system is systematically described in this paper. However, due to modeling size, the diffusion of oxygen atoms on the surface is not considered in this paper. In addition, in the future, we can also try to calculate the adsorption process without understanding the calculation method. Systematically compare the differences between different methods.

## Figures and Tables

**Table 1:** Structural parameters and energy data of the adsorption configuration of O_2_ on the surface of the plutonium gallium system.

Adsorption Conformation		Adsorption Energy *E*_ads_ (eV)	Oxygen Atomic Spacing *R_O_*_-*O*_ (Å)	Oxygen Atom to Surface Nearest Atom Distance *h_O_*_-*Pu/Ga*_ (Å)		Nearest Distance between Oxygen Atom and Surface *h_O_*_-*Surface*_ (Å)	
O-t	hor1	−6.0406	4.1317	2.1900	3.2502	0.6085	0.2281
	hor2	−8.5106	3.8721	2.1365	2.0500	0.4880	0.8873
	ver	−7.3165	2.7324	2.0540	2.2354	0.5599	0.1831
O-h	hor1	−9.6203	6.8671	2.1552	2.0726	0.9253	0.2106
	hor2	−9.4771	2.7048	2.1309	2.0189	0.1997	0.6141
	ver	−10.7000	4.0031	2.1887	2.1961	0.1108	0.2480
O-b	hor1	−10.3804	4.1748	2.3221	2.2002	1.0132	0.3981
	hor2	−6.9156	3.6822	2.0930	2.2135	0.0400	0.5042
	ver	−9.5051	3.3041	2.0941	2.1112	0.5968	0.4603

**Table 2:** Net charge distribution after optimization of each adsorption configuration.

Configuration		Adsorption of Atoms in Molecules			Total Number of Charges in Each Layer				
		Q_1_	Q_2_	Q_total_	Q_1st_	Q_2nd_	Q_3rd_	Q_4th_	Q_5th_
O_2_		0.0091	−0.0091	0.0000	--	--	--	--	--
Surface		--	--	--	−0.0050	−0.0377	0.1573	0.1915	−0.3062
H-t	hor1	1.2222	1.2258	2.4480	−2.4791	−0.0787	0.4194	0.0237	−0.3332
	hor2	1.1506	1.2245	2.3752	−2.4181	−0.3527	0.7470	0.0853	−0.4366
	ver	1.1762	1.2392	2.4154	−2.5363	−0.0398	0.4997	−0.0729	−0.2661
H-h	hor1	1.1303	1.2193	2.3496	−2.4283	−0.0315	0.4687	−0.0445	−0.3141
	hor2	1.2252	1.1877	2.4129	−2.3895	−0.1105	0.2109	0.1688	−0.2926
	ver	1.2271	1.2152	2.4424	−2.5452	−0.2250	0.8239	−0.0852	−0.4108
H-b	hor1	1.2509	1.2108	2.4617	−2.4698	−0.1225	0.5288	−0.1380	−0.2602
	hor2	1.2156	1.2373	2.4529	−2.1872	−0.3107	0.1202	0.1613	−0.2364
	ver	1.1336	1.1281	2.2618	−2.3769	0.1120	0.2726	−0.1081	−0.1614

**Table 3:** Variation of surface work function for different adsorption configurations (∆Φ).

Adsorption Conformation	Vacuum Energy Level/eV	Fermi Energy Level/eV	Power Letter/eV	∆Φ/eV
O-t-hor1	6.7774	3.8633	2.9141	−0.0755
O-t-hor2	6.9854	3.6591	3.3263	0.3368
O-t-ver	6.8152	3.8026	3.0126	0.0230
O-h-hor1	6.9134	3.6899	3.2235	0.2340
O-h-hor2	6.8247	3.8810	2.9437	−0.0458
O-h-ver	6.7524	3.7953	2.9571	−0.0325
O-b-hor1	6.7025	3.8454	2.8571	−0.1325
O-b-hor2	6.8617	3.9103	2.9514	−0.0381
O-b-ver	7.0312	3.5598	3.4714	0.4819
Surface	6.7817	3.7921	2.9896	--

## Data Availability

The study did not report any data.

## References

[1] Boring A.M., Smith J.L. (2000). Plutonium condensed-matter physics—A survey of theory and experiment. Los Alamos Sci..

[2] Ek J.V., Sterne P.A., Gonis A. (1993). Phase stability of plutonium. Phys. Rev. B Condens. Matter.

[3] Hecker S.S. (2008). Plutonium—An Element Never at Equilibrium. Metall. Mater. Trans. A.

[4] Shim J.H., Haule K., Kotliar G. (2007). Fluctuating valence in a correlated solid and the anomalous properties of δ-plutonium. Nature.

[5] Cooper N.G. (2000). Challenges in plutonium science. Los Alamos Sci. Los Alamos Sci. Lab..

[6] Schwartz A.J., Wall M.A., Zocco T.G., Wolfer W.G. (2005). Characterization and modelling of helium bubbles in self-irradiated plutonium alloys. Philos. Mag..

[7] Sadigh B., Wolfer W.G. (2005). Gallium stabilization of δ-Pu: Density-functional calculations. Phys. Rev. B.

[8] Luo W., Meng D., Li G., Huchi C. (2008). Electronic Structure and Formation Heat of Pu_3_M and PuM_3_ (M = Ga, In, Sn and Ge) Compounds. Acta Phys. Chim. Sin..

[9] Huda M.N., Ray A.K. (2004). Electronic Structures and Bonding of Oxygen on Plutonium Layers. Eur. Phys. J. B-Condens. Matter Complex Syst..

[10] Huda M.N., Ray A.K. (2005). Density functional study of O_2_ adsorption on (100) surface of γ-uranium. Int. J. Quantum Chem..

[11] Wei H., Song H., Xiong X., Wang G., Hu R., Luo S. (2009). Adsorption structure and electronic states of Oxygen atoms on δ-PU (111) surface. Comput. Appl. Chem..

[12] Guo J., Liu G., Wei H. (2013). First-principles study on ADSORPTION behavior of O_2_ on δ-Pu (100) Surface. Comput. Appl. Chem..

[13] Hernandez S.C., Venhaus T.J., Huda M.N. (2015). Atomic oxygen adsorption on 3.125 at.% Ga stabilized δ-Pu (1 1 1) surface. J. Alloy. Compd..

[14] Kresse G., Hafner J. (1993). Ab initio molecular dynamics for open-shell transition metals. Phys. Rev. B Cover. Condens. Matter Mater. Phys..

[15] Kresse G.G., Furthmüller J.J. (1996). Efficient Iterative Schemes for Ab Initio Total-Energy Calculations Using a Plane-Wave Basis Set. Phys. Review. B Condens. Matter.

[16] Blochl P.E. (1994). Projector augmented-wave method. Phys. Rev. B Condens. Matter.

[17] Kresse G., Joubert D. (1999). From ultrasoft pseudopotentials to the projector augmented-wave method. Phys. Rev. B.

[18] Perdew J.P., Burke K., Ernzerhof M. (1996). Generalized Gradient Approximation Made Simple. Physical Rev. Lett..

[19] Monkhorst H.J., Pack J.D. (1976). Special points for Brillouin-zone integrations. Phys. Review. B Condens. Matter.

[20] Bottin F., Jomard G., Geneste G. (2014). Water adsorption and dissociation on the PuO_2_(110) surface. J. Nucl. Mater. Mater. Asp. Fission Fusion.

[21] Hao Y.G., Eriksson O., Fernando G.W., Cooper B.R. (1993). Surface electronic structure of gamma -uranium. Phys. Rev. B Condens. Matter.

[22] Wei H., Hum R., Xiong X., Wang G., Song H., Luo S. (2010). Study on the first principle of H_2_ adsorption on δ-Pu (100) surface. Chin. J. Mol. Sci..

[23] Atta-Fynn R., Ray A.K. (2009). A first principles study of the adsorption and dissociation of CO_2_ on the δ-Pu (111) surface. Eur. Phys. J. B Condens. Matter Complex Syst..

[24] Henkelman G., Jόnsson H. (2000). Improved tangent estimate in the nudged elastic band method for finding minimum energy paths and saddle points. J. Chem. Phys..

[25] Henkelman G., Uberuaga B.P., Jόnsson H. (2000). A climbing image nudged elastic band method for finding saddle points and minimum energy paths. J. Chem. Phys..

